# Adult Movement Defects Associated with a CORL Mutation in *Drosophila* Display Behavioral Plasticity

**DOI:** 10.1534/g3.120.400648

**Published:** 2020-03-11

**Authors:** Agapi Dimitriadou, Nasia Chatzianastasi, Panagiota I. Zacharaki, MaryJane O’Connor, Samuel L. Goldsmith, Michael B. O’Connor, Christos Consoulas, Stuart J. Newfeld

**Affiliations:** * Medical School, National and Kapodistrian University of Athens, 11527 Athens, Greece; † Dept. Genetics, Cell Biology & Development, University of Minnesota, Minneapolis, MN 55455; ‡ School of Life Sciences, Arizona State University, Tempe AZ 85287

**Keywords:** ataxia, climbing, courtship, Fussel/SKOR, phototaxis

## Abstract

The CORL family of CNS-specific proteins share a Smad-binding region with mammalian SnoN and c-Ski protooncogenes. In this family Drosophila CORL has two mouse and two human relatives. Roles for the mouse and human CORL proteins are largely unknown. Based on genome-wide association studies linking the human CORL proteins *Fussel15* and *Fussel18* with ataxia, we tested the hypothesis that *dCORL* mutations will cause adult movement disorders. For our initial tests, we conducted side by side studies of adults with the small deletion *Df(4)dCORL* and eight control strains. We found that deletion mutants exhibit three types of behavioral plasticity. First, significant climbing defects attributable to loss of *dCORL* are eliminated by age. Second, significant phototaxis defects due to loss of *dCORL* are partially ameliorated by age and are not due to faulty photoreceptors. Third, *Df(4)dCORL* males raised in groups have a lower courtship index than males raised as singles though this defect is not due to loss of *dCORL*. Subsequent tests showed that the climbing and phototaxis defects were phenocpied by *dCORL^21B^* and *dCORL^23C^* two CRISPR generated mutations. Overall, the finding that adult movement defects due to loss of *dCORL* are subject to age-dependent plasticity suggests new hypotheses for CORL functions in flies and mammals.

Studies of a *dCORL* deletion (*fussel* in Flybase) revealed roles in TGF-β/Activin signaling during larval mushroom body development and in insulin expressing neurons in adults (Takaesu *et al.* 2102, Tran *et al.* 2018a,b). dCORL has two mouse relatives mCORL1 (mSKOR1) and mCORL2 (mSKOR2). mCORL1 transcription in embryos is found only in dorsal interneurons of the cerebellum (Mizuhara *et al.* 2005), while mCORL2 is expressed only in Purkinje neurons of the cerebellum (Minaki *et al.* 2008). *mCORL2* knockout studies revealed a requirement for Purkinje cell differentiation via the inhibition of interneuron fate (Miyata *et al.* 2010; Wang *et al.* 2011; Nakatani *et al.* 2014). No *mCORL1* knockout has been reported. In adults *mCORL1* is primarily, though not exclusively, expressed in the cerebellum while *mCORL2* is restricted to the cerebellum (Yue *et al.* 2014). In adults, the specific neurons expressing the *mCORL* genes are unknown. A recent study of *mCORL* transgenes in Drosophila demonstrated that mCORL2 and dCORL have a common function with mCORL1 fulfilling a distinct function (Stinchfield *et al.* 2019).

dCORL has two human relatives *Fussel15* (hSKOR1) and *Fussel18* (hSKOR2). RT-PCR studies report that the *Fussel15* expression pattern is the same as its homolog *mCORL1*, primarily though not exclusively in the adult cerebellum. The *Fussel18* expression pattern is the same as its homolog *mCORL2*, restricted to the adult cerebellum (Fagerberg *et al.* 2014). Genome-wide association studies have linked single nucleotide polymorphisms in *Fussel15* to three ataxias - Essential Tremor, Periodic Leg Movement and Restless Leg (Kemlink *et al.* 2009; Moore *et al.* 2014; Li *et al.* 2017; Chen *et al.* 2018). An association of *Fussel18* with ataxia has also been reported (Valence *et al.* 2019). Ataxias are movement disorders arising from dysfunction in the cerebellum, where *Fussel15* and *Fussel18* are expressed.

Taking cues from: 1) the study of the *mCORL2* knockout in Pukinje neurons of the cerebellum, 2) *Fussel15* and *Fussel18* associations with ataxia, 3) the demonstration that *dCORL* functions in adult brains and 4) the similarity of transgenic *mCORL2* and *dCORL* functions, we examined *dCORL* mutant adults for movement defects. Our hypothesis was that the association of *Fussel15* and *Fussel18* with ataxia will be conserved and visible in *dCORL* adult mutants as defects in one or more movement-based behaviors.

Here we report adult behavioral studies of the small deletion *Df(4)dCORL* and eight control strains. We found that *Df(4)dCORL* flies exhibit three types of behavioral plasticity. First, significant climbing defects attributable to loss of *dCORL* are eliminated by age. Second, significant phototaxis defects due to loss of *dCORL* are partially ameliorated by age and are not due to faulty photoreceptors. Third, *Df(4)dCORL* males raised in groups have a lower courtship index than males raised as singles though this defect is not due to loss of *dCORL*. Subsequent tests showed that the climbing and phototaxis defects were phenocpied by *dCORL^21B^* and *dCORL^23C^* two CRISPR generated mutations. Overall, the finding that adult movement defects due to loss of *dCORL* are subject to age-dependent plasticity suggests new hypotheses for CORL functions in flies and mammals.

## Materials and Methods

### Fly stocks

The *Df(4)dCORL* parental insertions *Pbac{WH}f07015* and *Pbac{WH}f06253* are as described (Takaesu *et al.* 2012). The null allele *sphinx^720RW^* is as described (Dai *et al.*, 2008). The *Pbac{RB}e02096* insertion disrupts all predicted transcripts of the eIF4E-Transporter (CG32016), a gene whose primary expression is in oogenesis (Flybase; Thurmond *et al.* 2019). The P excision alleles *Glu-RA^112B^* (a large deletion; noted here as *Glu-RA-null*) and *Glu-RA^2B^* (a precise excision reverting to wild type; noted here as *Glu-RA-control*) are as described (Bogdanik *et al.* 2004). The genomic positions of these lines in relation to the only functional *dCORL* start site (Tran *et al.* 2018a) is in Figure S1. Canton-S, *yw* and *w^1118^* are common stocks in fly labs. Two *dCORL* CRISPR mutant lines and a line that went through CRISPR mutagenesis but has a wild type *dCORL* sequence are described below.

### dCORL CRISPR mutagenesis

Guide RNAs for *dCORL* were designed using the CRISPR Optimal Target Finder (http://targetfinder.flycrispr.neuro.brown.edu/) using the following parameters: guide length 20 base pairs, 5′GG only. Oligos to targets 1 and 4 were cloned into pU6 BbsI chi RNA resulting in two gRNAs that were co-injected by BestGene Inc. (Chino Hills, CA) into embryos carrying maternally expressed Cas9 (PBac{y[+mDint2]=vas-Cas9}VK00027; BDSC #51324). Thirty-nine G0 flies were mated to flies from a transheterozygous 4^th^ chromosome stock (P{w[+mC]=ActGFP}unc-13[GJ]/In(4)ci[D]; BDSC #9549). After successful mating, G0 flies were screened for deletions by PCR analysis using the target 4 forward and target 1 reverse oligos that flank both targeted sites. Five G0 lines yielded additional fragments, suggestive of a deletion. From these lines, 8 single males carrying a putative dCORL deletion over ci^D^ were crossed to parental females and rescreened by PCR for the presence of a deletion. Offspring from two of the five G0 founders yielded independent and deletions in the Sno homology domain of exon 3 (*dCORL^21B^* and *dCORL^23C^*). These were confirmed by sequencing homozygotes. A line that went through the same process but showed no errors in *dCORL* was kept as a control for off-target phenotypes (*dCORL^4^*).

gRNA oligos:

dCORL target 1 for 5′P-CTTCGGTGTTCCCTTTAACTTACA-3′

dCORL target 1 rev 5′P-AAACTGTAAGTTAAAGGGAACACC-3′

dCORL target 4 for 5′P-CTTCGCGTAATCATGCCACATCGT-3′

dCORL target 4 rev 5′P-AAACACGATGTGGCATGATTACCC-3′

PCR and sequencing oligos

dCORL target 1 rev 5′-GTTTGAGTCTGGTTTAACCCAG-3′

dCORL target 4 for 5′-GGAGTGCAGATTGTATCCCTG-3′

### Behavior assays

#### Adult climbing:

20 males or 20 females at 5 or 10 days old were examined individually in 14 ml tubes with an interior coating of propylene that facilitates climbing (Falcon). Every fly was tested six times in two rounds of three trials at a temperature of 22° and humidity greater than 60%. The Falcon tubes were marked at 6cm from the bottom and the test was to estimate the climbing time for the 6cm distance. If a fly failed to complete the test within ten seconds, then that was considered a failure. From this experiment three parameters were estimated. 1) The average climbing time (average score) per genotype was estimated by finding the average time of each fly (average of six trials) and then calculating the overall average scores of all flies. 2) The average best score was calculated by averaging the best score (the shortest climbing time from six trials/fly) of all flies per genotype. 3) The average failure rate was calculated by the same methods as average time across all individuals of a given genotype.

#### Adult phototaxis:

20-30 males or females at 5 or 10 days old were transferred into 25ml sterilized plastic pipettes with both sides blocked by cotton. The pipette was then placed vertically on the surface of the bench top causing the flies to be concentrated at the lower end. Then the pipette was placed horizontally and the end opposite to the flies was illuminated. After 30 sec the other end of the pipette was illuminated. This procedure was repeated fifteen times. The number of flies crossing the midline of the pipette within fifteen seconds by moving either toward or away from the light source was counted.

#### Adult male courtship:

Five day old males were tested. In the “singles” assays, males were collected as late pupae and isolated in vials (one fly per vial) until the age of 5 days. Each individual was examined for courtship with a virgin female of same age and genotype (between 10 and 25 males were examined for each genotype). In the “group” assays, 10 to 25 males were collected as late pupae and placed together in the same vial. When the group reached 5 days old, each individual was examined for courtship with a virgin female of same age and genotype. All virgin females were stored in groups before testing (20 females per vial). The assay was conducted with one male and one virgin female in a courtship chamber (1.2cm diameter × 0.8cm high), which contained a layer of yeast media. Courtship index is the percentage of time that the male spent successfully courting the female during a 10 min period (*e.g.*, orientation, vibrating wings and attempting to copulate).

#### Larval crawling:

Five groups of ten early third instar larvae were collected, washed in dH_2_O and placed at the center of an agar-coated 10cm petri dish. One minute videos of spontaneous crawling activity were recorded and the average and best scores estimated from bouts of continuous crawling. There were a total of 50 videos for each genotype. A template at the bottom of the dish (2mm of increasing diameter for seven concentric circles) was used to facilitate measurements of the distance traveled. At the end of a minute a snapshot was taken and the distribution of the larvae quantitated via the concentric circles.

### Electroretinogram recordings

Recordings were collected from 10 day old male and female *Df(4)dCORL* (n = 12) and *Glu-RA-control* (n = 8) flies at 22° and 40–70% relative humidity as described (Wu and Wong 1977). Electrodes were created from filamented glass micropipettes (1.2 mm OD, 0.8 mm ID, WPI, Sarasota, FL) with an electrode-puller (P97 Flaming/Brown Micropipette Puller, Shutter Inst.). Electrodes were filled with a saline solution (0.7% w/v NaCl) and inserted into an electrode holder containing a chloridized silver wire. The electrode resistance was ∼10 MΩ. The recording electrode was inserted into the cornea, while the ground electrode, filled with 3M NaCl, was inserted into the proboscis. Signals were recorded with a DC amplifier (Axoclamp-2B, Molecular Devices). The signals were sampled at 10 kHz by a data acquisition device (Digidata 1200, Molecular Devices) and without filtering were analyzed and displayed with Clampex v8.1 software (Molecular Devices). A generic cool-white LED driven at a constant voltage delivered light flashes. The LED was positioned toward the eye, approximately 1 cm away, and every fly received three light flashes of 500 ms separated by 60 sec of dark adaptation. Means for each genotype were compared with an unpaired, two-tailed Student’s T-test.

### Longevity assays

Measurement of the lifespan of males and females, as virgins and after mating, was conducted as described (Tran *et al.* 2018b).

### Statistical analysis

**Figure 1:**
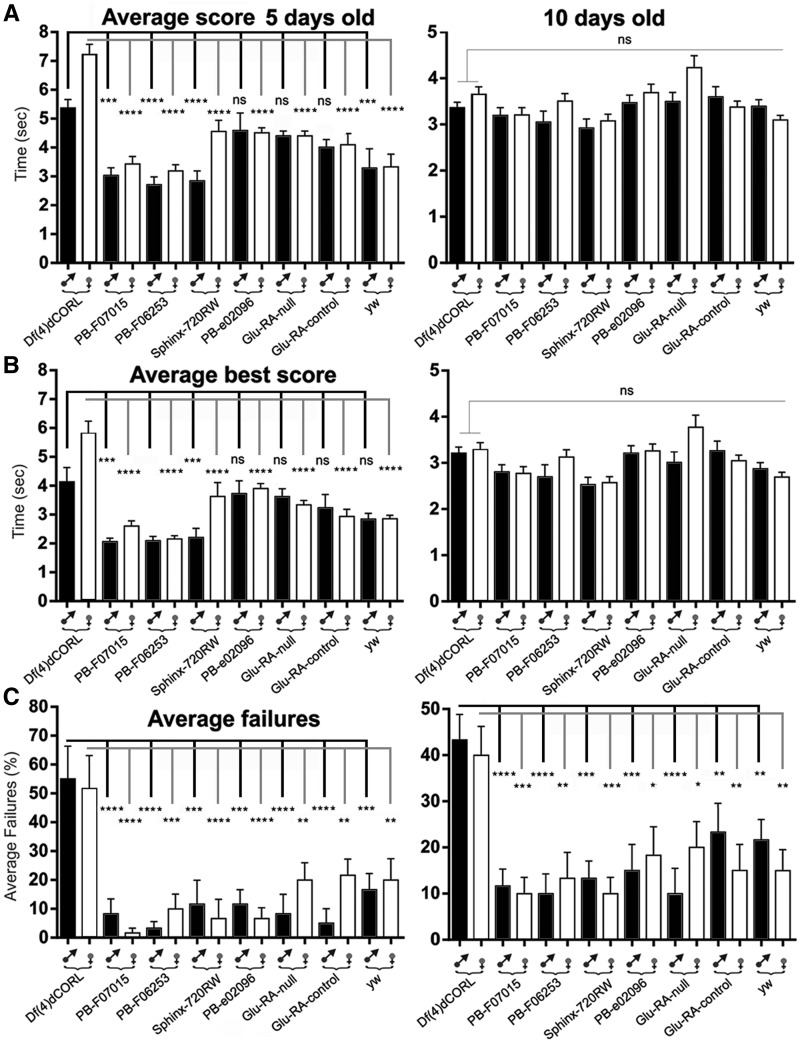
Climbing defects in *Df(4)dCORL* adults display age-dependent plasticity attributable to *dCORL*. A-C) Climbing assays for 5 day (left side) and 10 day old males and females analyzed separately from eight strains: from left *Df(4)dCORL*, its parental lines *Pbac{WH}f07015* and *Pbac{WH}f06253*, four lines associated with lesions in the other three genes in the deletion and *yw* as a control for the genetic background of *Df(4)dCORL*. A genomic map identifying the location of the relevant mutation in each strain (except yw) is in Fig. S1. Bar graphs in the top two rows display the mean of elapsed time (extending bars report standard error), while the bottom row shows the mean number of incomplete trials for each genotype. Numerical data for 5 day olds in Table S1 and 10 day olds in Table S2. A) Average score (average time to climb a 6cm vertical distance) for 5 day olds. *Df(4)dCORL* females have a significantly slower average speed than all others, while males are significantly slower than 4 lines and slower but not significantly than 3 lines. Average score for 10 day olds. *Df(4)dCORL* males and females have increased their average speed and are not distinguishable from all others, while all others have maintained the same average speed. B) Average best score (the minimum time to climb a 6cm vertical distance) for 5 and 10 day olds show exactly the same pattern. *Df(4)dCORL* females have a significantly slower maximum speed than all others while males are significantly slower than 4 lines and slower but not significantly than 3 lines. Average best score for 10 day olds. *Df(4)dCORL* males and females have increased their top speed and are not distinguishable from all others, while all others have maintained the same top speed. C) Average failure rate (percentage of trials an individual fails to cover the 6cm distance within ten seconds) for 5 day olds. *Df(4)dCORL* males and females have a significantly higher failure rate than all the others. Average failure rate for 10 day olds. All flies reduced their failure rate by roughly the same amount leaving *Df(4)dCORL* males and females with a significantly higher failure rate. Significant results are noted as * *P* < 0.05, **P < 0.01, ***P < 0.001, ****P < 0.0001 with nonsignificant (ns) *P* > 0.05.

**Figure 2:**
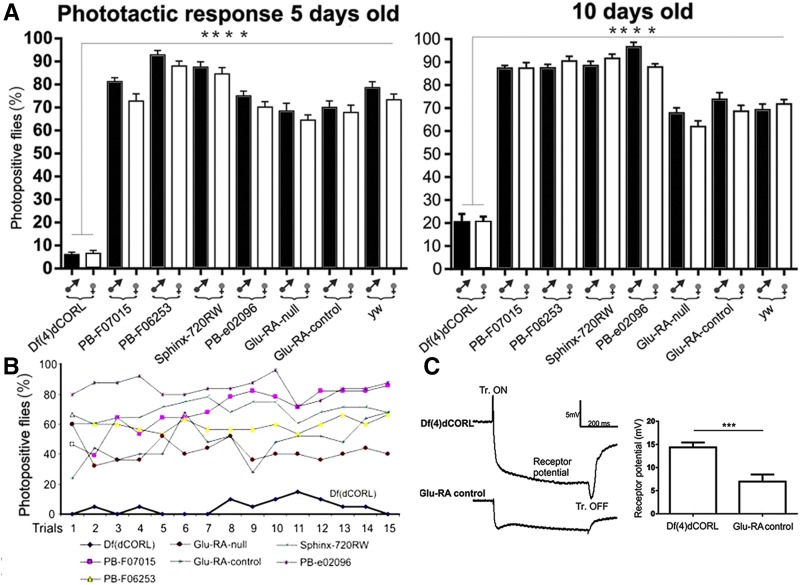
Phototaxis defects in *Df(4)dCORL* adults display age-dependent plasticity attributable to dCORL but are not due to faulty photoreceptors. A) Phototaxis assay of 5 day and 10 day old males and females from 8 strains. Graphs report the percent of flies of each genotype that move toward light when given a binary choice. Numerical data in Table S3. Phototaxis assays reveal an effect of age on performance similar to that seen with climbing. 10 day old *Df(4)dCORL* flies are significantly improved compared to 5 day olds. For the other strains no obvious pattern is visible with some strains improved with age while others decreased. The distinction between *Df(4)dCORL* and all other strains at both ages was highly significant. B) Detailed results from the assay of 5 day old males. Of fifteen trials with *Df(4)dCORL*, six had no *Df(4)dCORL* flies move toward the light. In contrast males of the same age in all other lines show strongly phototactic responses in all trials. C) Electroretinograms from *Df(4)dCORL* and *Glu-RA-control* males. Receptor potential (measured in millivolts) in *Df(4)dCORL* flies is significantly more robust than in the *Glu-RA-control*. The contrast between *Df(4)dCORL* and *Glu-RA-control* in the phototaxis (*Df(4)dCORL* worse) and retinogram assays (*Df(4)dCORL* better) indicates that the phototaxis defect in *Df(4)dCORL* is not due to a photoreceptor deficit.

**Figure 3:**
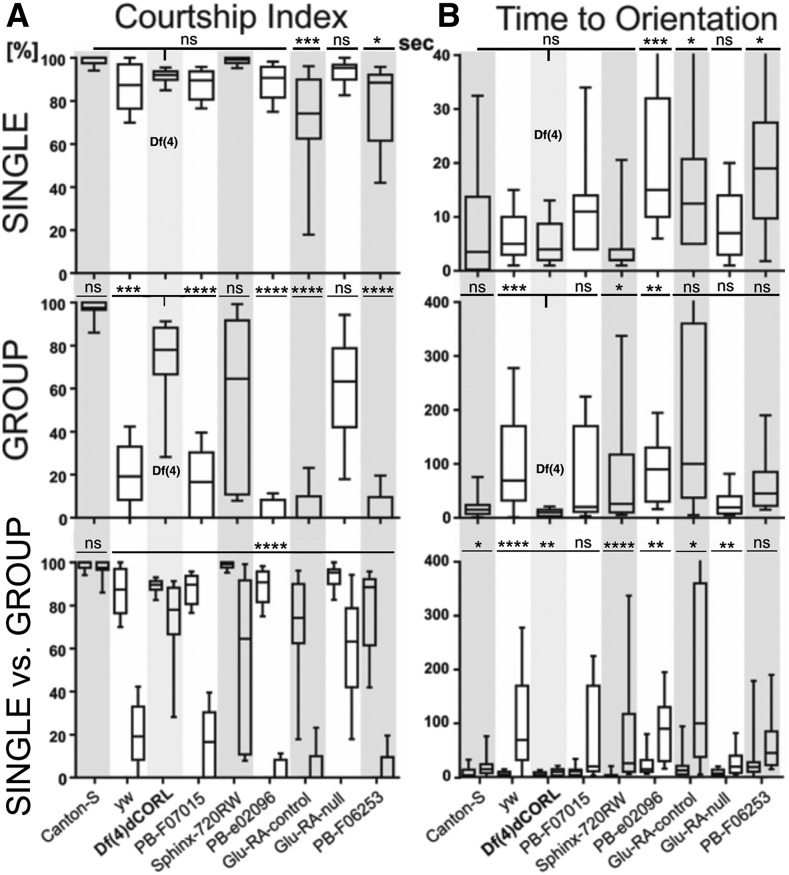
*Df(4)dCORL* defects in male courtship are not attributable to loss of *dCORL*. Column A) Courtship index is the percent of time that a 5 day old male spends successfully courting during a 10 min interval. Top row Single. Males from 8 strains that were raised in isolation from the pupal stage. Males from 6 of the strains including *Df(4)dCORL* courted 5 day old congeneric virgin females at wild type levels as represented by Canton-S. Two strains spent less time courting. Numerical data in Table S4. Middle row Group. Males were raised in groups of 10-25 from eclosion. Males from 3 of the strains including *Df(4)dCORL*, courted 5 day old congeneric virgin females at wild type levels. Four strains spent less time courting. Bottom row Single *vs.* Group. Males from all 8 strains, including *Df(4)dCORL* showed significant reductions in courtship index when raised in groups *vs.* when raised as singles, but not Canton-S. Column B) Orientation is the time it takes for a 5 day old male to orient its body toward a 5 day old congeneric virgin female (representing courtship initiation). Top row Single. Males from five strains including *Df(4)dCORL* oriented toward 5 day old congeneric virgin females at wild type levels. Three strains took more time to orient themselves. Middle row Group. Males from five strains including *Df(4)dCORL* oriented toward 5 day old congeneric virgin females at wild type levels. Bottom row Single *vs.* Group– Males of all strains including *Df(4)dCORL* took longer to orient themselves in groups then when raised as singles, including Canton-S.

In Figures 1,2,6,7 data are presented in bar graphs representing the mean with extending bars indicating the standard error of the mean. In Figure 3, 4 data are presented as box blots representing the median, 25^th^ and 75^th^ percentiles with extending bars indicating the 10^th^ and 90^th^ percentiles. Initial results were evaluated via Graphpad6 software with D’Agostino-Pearson omnibus normality tests. Results passing the normality test were evaluated by non-parametric Kruskal-Wallis one-way ANOVA followed by Dunn’s multiple comparison tests. When the data did not pass the normality test then they were evaluated by an ordinary one-way ANOVA followed by Tukey’s multiple comparison tests. Statistical significance in graphs and tables is noted as follows: * *P* < 0.05, ** *P* < 0.01, *** *P* < 0.001, ****P < 0.0001 and not significant (ns) *P* > 0.05.

### Data availability

Strains are available upon request. The authors affirm that all data necessary for confirming the conclusions of the article are present within the text, figures and supplemental materials. Supplemental material available at figshare: https://doi.org/10.25387/g3.11968362.

## Results

### Adult movement defects attributable to loss of dCORL show age-dependent plasticity

In two previous studies we employed six control strains to determine if a phenotype observed for *Df(4)dCORL* was attributable to the loss of *dCORL* and not one of the other three genes in the deletion (Takaesu *et al.* 2012, Tran *et al.* 2018b). Two of the six are the parental PiggyBac insertions employed to generate *Df(4)dCORL* and four lines carry mutations in the other three genes. Tested alleles for two of the genes were previously shown to be nulls: *Glu-RA-null* is a deletion resulting from an imprecise excision and *spx^720RW^* is an engineered mutation disabling splicing. For the third gene, *Pbac{RB}e02096* is predicted to disrupt all transcripts of the eIF4E-Transporter and should also be a null (Fig. S1). We showed that *Df(4)dCORL* does not affect the adjacent *twin** of eyeless* (Takaesu *et al.* 2012, Tran *et al.* 2018b). We employed these six strains, plus yw as a control for genetic background, in studies of *Df(4)dCORL* adult behavior (8 lines total).

The project began with a climbing assay in 5 day old adults of both sexes separately for *Df(4)dCORL* and the seven control lines. In Figure 1A we show the average score (climbing time) and in Figure 1B the average best score (fastest climbing time). *Df(4)dCORL* females require significantly longer time to complete the task than all other lines in both measures. *Df(4)dCORL* males require more time than all strains but are only significantly slower than 4 of them. We observed that *Df(4)dCORL* flies exhibit longer climbing times because they stop frequently and wander horizontally rather than climbing vertically. In Figure 1C we show that *Df(4)dCORL* flies fail to complete the climbing test signficantly more often than all other flies of both sexes. The results of these assays suggest that the climbing defect of *Df(4)dCORL* flies is attributable to loss of *dCORL*. Numerical data are shown in Table S1.

To ascertain if there was any age-dependent plasticity in the *Df(4)dCORL* climbing phenotype we examined 10 day old males and females separately. It is easily visible in Figure 1A,B that age significantly improved both climbing average score and average best score of *Df(4)dCORL* flies from those of 5 day old mutants. As none of the control strains showed any difference with age, both scores for 10 day old *Df(4)dCORL* now match all controls in both sexes. Age-dependent plasticity is also seen for the climbing failure rate of *Df(4)dCORL* flies shown in Figure 1C. There is improvement of roughly 10% in the failure rate strictly due to age. However, this remained significantly higher than in all the other lines, which did not show improvement. As a result, *Df(4)dCORL* did not catch up as they did in climbing time. The results demonstrate that the *dCORL* mediated climbing defects in speed and completion of *Df(4)dCORL* flies are subject to age-dependent plasticity. Numerical data are shown in Table S2.

Next we deployed phototaxis assays examining the sexes separately from the same eight strains. Figure 2A shows that *Df(4)dCORL* flies of both sexes were significantly impaired in their light response. The average number of photopositive *Df(4)dCORL* flies was roughly 10% the number in all other lines. As shown in Figure 2B, for six of the fifteen trials no *Df(4)dCORL* flies moved toward the light. This was not seen in any other line in any trial. We observed that *Df(4)dCORL* flies initially react positively but then wander randomly reminiscent of their climbing phenotype. The results of these assays suggest that the light response defect of *Df(4)dCORL* flies is attributable to loss of *dCORL*. Numerical data are shown in Table S3.

Examining 10 day old *Df(4)dCORL* flies for phototaxis defects again revealed age-dependent plasticity. *Df(4)dCORL* males and females consistently performed better than 5 day olds (Figure 2A). As with climbing failures, even though the results for the other lines did not change *Df(4)dCORL* did not catch up. This line still contained significantly fewer photopostive flies. The results demonstrate that the *dCORL* mediated defects in light response of *Df(4)dCORL* flies are subject to age-dependent plasticity. Overall we observed in the climbing and phototaxis assays that *Df(4)dCORL* flies initiate normally but are unable to sustain either behavior due to the loss of *dCORL*. The climbing and phototaxis data for *Df(4)dCORL* displayed age-dependent plasticity with improvement in all 4 tests. Improvement was sufficient to erase the climing speed defects but not the completion or phototaxis defects.

To address the hypothesis that the phototaxis defect in *Df(4)dCORL* flies was due to a vision defect, rather than a motor defect, we compared electrinoretinograms of *Df(4)dCORL* 10 day old males with the same age males from one of the control lines. We chose the *Glu-RA-control* strain that contains a precise excision reverting to wild type at the sequence and phenotype levels. We found that photoreceptor potential (as measured in millivolts) in *Df(4)dCORL* flies is significantly more robust than in the *Glu-RA-control* (Figure 2C). Contrast between *Df(4)dCORL* and *Glu-RA-control* performance in the phototaxis assay (Figure 2A with *Df(4)dCORL* significantly worse than *Glu-RA-control*) and the retinogram assay (Figure 2C with *Df(4)dCORL* significantly better than *Glu-RA-control*) indicates that the phototaxis defect in *Df(4)dCORL* flies is not due to a vision deficit. Numerical data in Table S3. The data support our initial hypothesis that *dCORL* adult mutants will display movement-based defects.

### Df(4)dCORL defects in male courtship are not attributable to loss of dCORL

**Figure 4:**
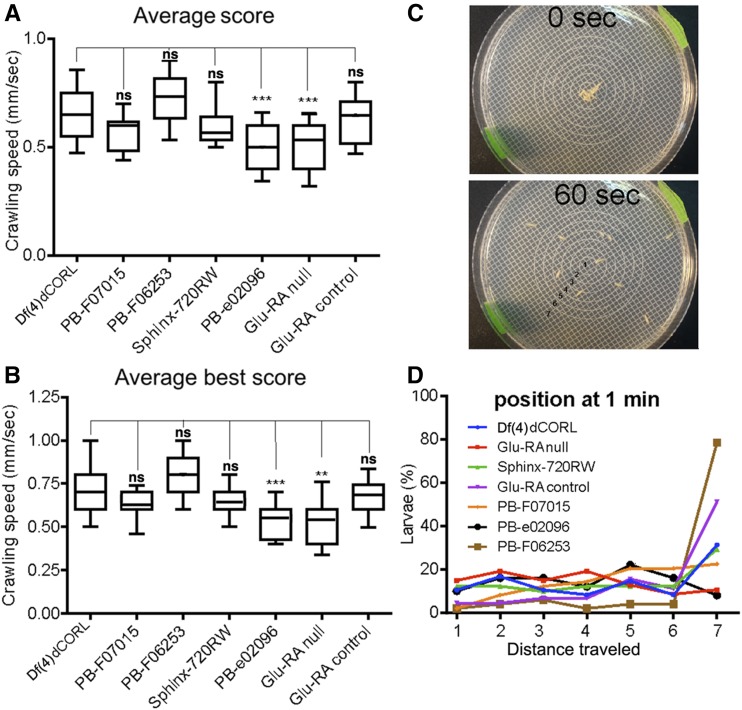
*Df(4)dCORL* larvae do not display defects in crawling speed or crawling distance. A,B) Average score and average best score for crawling speed of *Df(4)dCORL* larvae and six control strains. *Df(4)dCORL* is not significantly different from four strains but *Pbac{RB}e02096* and *Glu-RA-null* larvae are significantly slower than *Df(4)dCORL*. Numerical data in Table S6. C) Representative snapshot of the larval distance traveled assay at time 0 and 60 sec. D) Frequency distributions of distance traveled after 60 sec of crawling for all strains. No significant differences in distance traveled were detected. Numerical data in Table S7.

We next turned to courtship assays to determine if *Df(4)dCORL* males displayed a phenotype attributable to loss of *dCORL* and if so, whether there was any behavioral plasticity. In this case we examined social context dependent plasticity in the courtship index (the percent of time spent in courtship leading to mating success within a ten minute period) for 5 day old virgin males with a virgin female of the same age and genotype. We added an additional control strain, the true wild type Canton-S. For males raised singly from eclosion to 5 days old, *Df(4)dCORL* was not different than Canton-S. There was a significant reduction in courtship in 2 strains compared to *Df(4)dCORL* (Figure 4A top). For males raised and mated in groups, *Df(4)dCORL* was not different than Canton-S. There was a significant reduction in courtship index for 5 strains compared to *Df(4)dCORL* (Figure 4A middle). When looking within genotypes at the courtship of singles *vs.* group raised males, all strains including *Df(4)dCORL* showed significant reductions in group raised males but not Canton-S. Numerical data in Table S4.

Interestingly, the median for the courtship index of *Df(4)dCORL* males raised in groups was the highest of all strains in this test except for Canton-S. Males from the parental line *Pbac{WH}f06253* and from *Pbac{RB}e02096* were the worst at group courtship completion. Curiously *Glu-RA-null* males performed better than *Glu-RA-control* males. Taken together the: 1) insignificant difference of single and group raised male courtship for *Df(4)dCORL* and Canton-S, 2) strength between group raised male courtship for *Df(4)dCORL* *vs.* other mutant strains, 3) unexpectedly strong performance of group raised male courtship for *Glu-RA-null* *vs.* *Glu-RA-control* and 4) the poor performance of group raised male courtship for *Pbac{WH}f06253* and *Pbac{RB}e02096* suggest that the group raised male courtship defect in *Df(4)dCORL* was not due to loss of *dCORL*.

To determine if the reduction in courtship index for group raised males was due to failure to initiate or failure to complete courtship, the initial step of courtship (time to orientation) was evaluated for social context dependent plasticity. For males raised singly *Df(4)dCORL* was not different than Canton-S. There was a significant increase in time to orientation for 3 strains compared to *Df(4)dCORL* (Figure 4B top). Males raised and mated in groups on average took an order of magnitude longer to orient than their single counterparts in all strains. *Df(4)dCORL* was not different than Canton-S. There was a significant increase in time to orientation for 3 strains compared to *Df(4)dCORL* (Figure 4B middle). When looking within genotypes at the time to orientation of singles *vs.* group raised males, all strains (including Canton-S and *Df(4)dCORL)* showed significant increases with 2 exceptions. Six strains showed increases in time to orientation and reductions in courtship index when comparing males raised as singles to those raised in groups. We conclude that males when raised in groups fail to begin courtship normally resulting in a reduced courtship index. Numerical data in Table S5.

Overall, the effects of social context on *Df(4)dCORL* time to orientation or courtship index are not attributable to loss of *dCORL*. As courtship behavior is an innate motor behavior (like climbing and phototaxis), the data on courtship reveal that not all innate behavioral phenotypes of *Df(4)dCORL* flies are *dCORL* related

### Df(4)dCORL larvae do not display defects in crawling speed or distance traveled

To ascertain whether the defects in adult climbing were due to defects in development (manifest in larvae and persist in adults) or physiology (only manifest in adults), we examined larval crawling average speed, maximum speed and distance traveled. We tested *Df(4)dCORL* and the six original control lines. In Figure 4A we show the average score (average crawling speed) and in Figure 4B the average best score (maximum crawling speed). We found that two lines were slower than all other strains in average score and average best score. These were *Pbac{RB}e02096* and *Glu-RA-null*. *Df(4)dCORL* was not significantly different than the other four lines. Numerical data in Table S6. We measured crawling ability as distance traveled in 60 sec (example in Figure 4C). In this assay *Df(4)dCORL* larvae were not significantly different from any of the other strains (Figure 4D). Numerical data in Table S7. Overall, larval crawling data shows that the climbing defects of *Df(4)dCORL* adults are not developmental but are instead due to errors in adult physiology.

### dCORL^21B^ and dCORL^23C^ phenocopy Df(4)dCORL adult climbing and phototaxis defects

**Figure 5:**
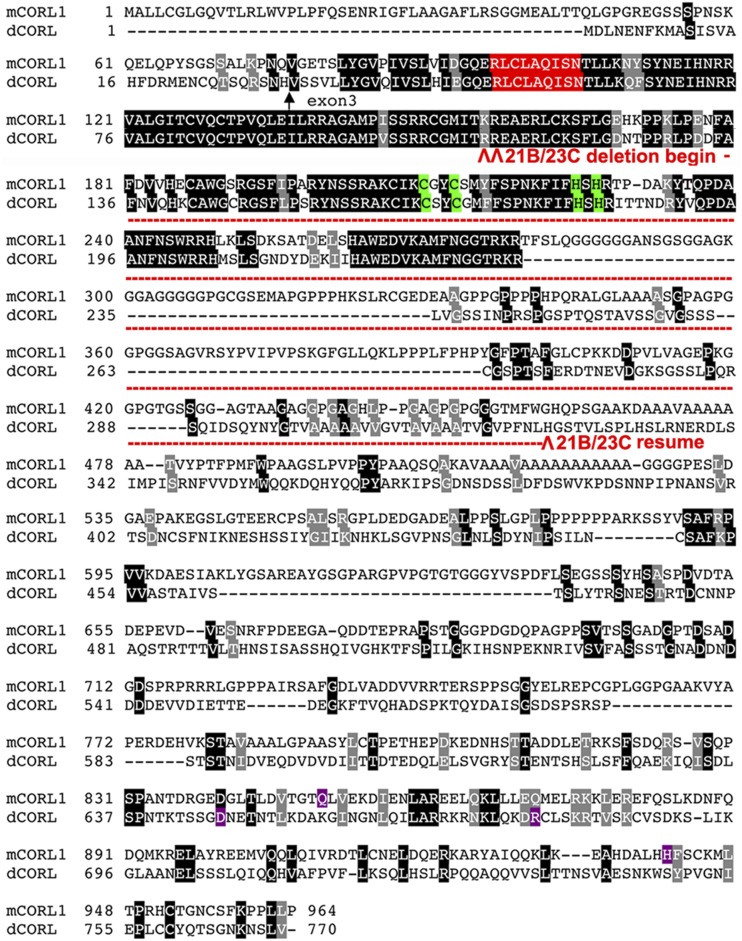
Loss of the majority of the Sno homology domain in two dCORL CRISPR mutants. An alignment between mCORL1 and dCORL is shown. A black arrow indicates the splice acceptor site for dCORL exon3. The highly conserved Sno homology domain begins six amino acids downstream. The starting points for two CRISPR induced deletions in adjacent Arginine residues within the Sno homology domain are shown with red arrowheads. In both mutations the Zinc finger domain (green) is lost while the APC recognition site that allows a protein to be marked by ubiquitylation for degradation is intact. *dCORL^21B^* and *dCORL^23C^* are deletions of 218 or 217 amino acids respectively beginning at Arg^104^ or Arg^105^ and ending at His^322^. The deletions remove 62% of the Sno homology domain. DNA sequence data for these mutations are shown in Fig. S2.

To be sure that the climbing and phototaxis defect seen in *Df(4)dCORL* adults were due to loss of *dCORL* and not another gene in the deletion or a synergystic effect of the loss of multiple genes we generated mutations in *dCORL* by CRISPR. Two independent mutations were identified. A non-mutant line that went through the CRISPR process (*dCORL^4^*) was kept to serve as a control for off target effects. *dCORL^21B^* and *dCORL^23C^* are large in-frame deletions of 218 or 217 amino acids respectively starting in the highly conserved Sno homology domain (mutant amino acid sequences Figure 5 and mutant nucleotide sequences Fig. S2). Specifically, *dCORL^21B^* removes amino acids between Arg^104^ and His^322^, while *dCORL^23C^* removes amino acids between Arg^105^ and His^322^ (numbering from GenBank JX126878.1). In both the Zinc finger domain is lost while the APC recognition site that allows a protein to be marked by ubiquitylation for degradation is intact. The deletions remove 62% of the Sno homology domain and likely create null alleles.

**Figure 6:**
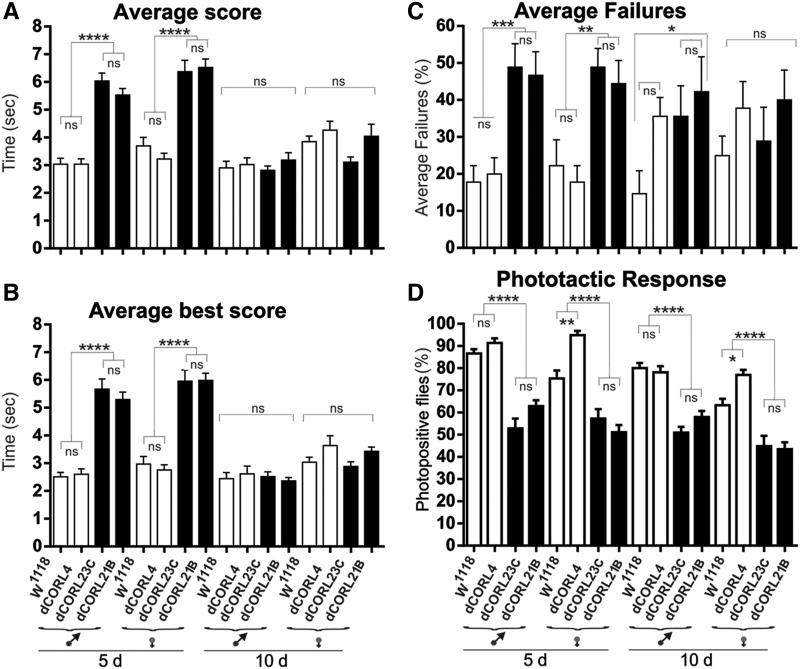
*dCORL^21B^* and *dCORL^23C^* phenocopy *Df(4)dCORL* adult climbing and phototaxis defects. A-C) Climbing assays for adults at ages 5 and 10 days old, separated by sex from four strains: *w^1118^* parental, *dCORL^4^* CRISPR control and two CRISPR deletion mutants *dCORL^23C^* and *dCORL^21B^*. Numerical data in Tables S8-S10. A) Average score shows that *dCORL^23C^* and *dCORL^21B^* males and females are significantly slower than *w^1118^* and *dCORL^4^* at 5 days old. At 10 days old there is no difference between the strains for either sex. This is the same result as *Df(4)dCORL* in Fig. 1A. B) Average best score shows that *dCORL^23C^* and *dCORL^21B^* males and females are significantly slower than *w^1118^* and *dCORL^4^* at 5 days old. At 10 days old there is no difference between the strains for either sex. This is the same result as *Df(4)dCORL* in Fig. 1B. C) Average failure rate shows that *dCORL^23C^* and *dCORL^21B^* males and females do not complete the climb significantly more often than *w^1118^* and *dCORL^4^* at 5 days old. At 10 days there is no difference between the strains for females as seen for *Df(4)dCORL*. *dCORL^23C^* and *dCORL^21B^* males improve but do not quite match the *w^1118^* completion rate at the lowest level of significance. This is the same result as *Df(4)dCORL* males in Fig. 1C. D) Phototaxis assays show that *dCORL^23C^* and *dCORL^21B^* males and females have a significantly weaker response to light than *w^1118^* and *dCORL^4^* at 5 days old and this defect persists at 10 days old. This is the same result as *Df(4)dCORL* in Fig. 2A.

We tested homozygous escaper males and females at 5 and 10 days old from *dCORL^21B^*, *dCORL^23C^* and *dCORL^4^* for climbing and phototaxis defects. We employed the *w^1118^* parental line as a further control. In the first climbing assay, average score, we found that *dCORL^23C^* and *dCORL^21B^* males and females are significantly slower than *w^1118^* and *dCORL^4^* at 5 days. The two control lines were not different from each other nor were the two mutant lines. At 10 days there is no difference between any of the strains for either sex (Figure 6A, numerical data Table S8). The age-dependent plasticity for climbing average speed is the same result seen for *Df(4)dCORL* males and females as shown in Figure 1A. The second climbing assay, average best score, shows the same pattern as average score (Fig. 6B, Table S9). The CRISPR mutants match the results for *Df(4)dCORL* (Figure 1B).

The third climbing assay, average failure rate, shows the same results as both speed assays for *dCORL^23C^* and *dCORL^21B^* females at 5 and 10 days. The mutant females caught up to the controls. Alternatively, mutant males catch up to *dCORL^4^* but not quite to *w^1118^* (Figure 6C, Table S10). Reduction in failures for both sexes was also seen for *Df(4)dCORL* (Figure 1C). However, mutant males’ inability to catch up is similar to *Df(4)dCORL* but females’ ability to catch up to the others is not. In the phototaxis assay *dCORL^23C^* and *dCORL^21B^* males and females have a significantly weaker response to light than *w^1118^* and *dCORL^4^* at 5 days and this defect persists at 10 days (Figure 6D, Table S11). These results are the same as seen for *Df(4)dCORL* (Figure 2A). Overall in 7 of 8 behavioral studies (males and females in four assays) *dCORL^23C^* and *dCORL^21B^* match the results for *Df(4)dCORL*, including age-dependent plasticity.

**Figure 7:**
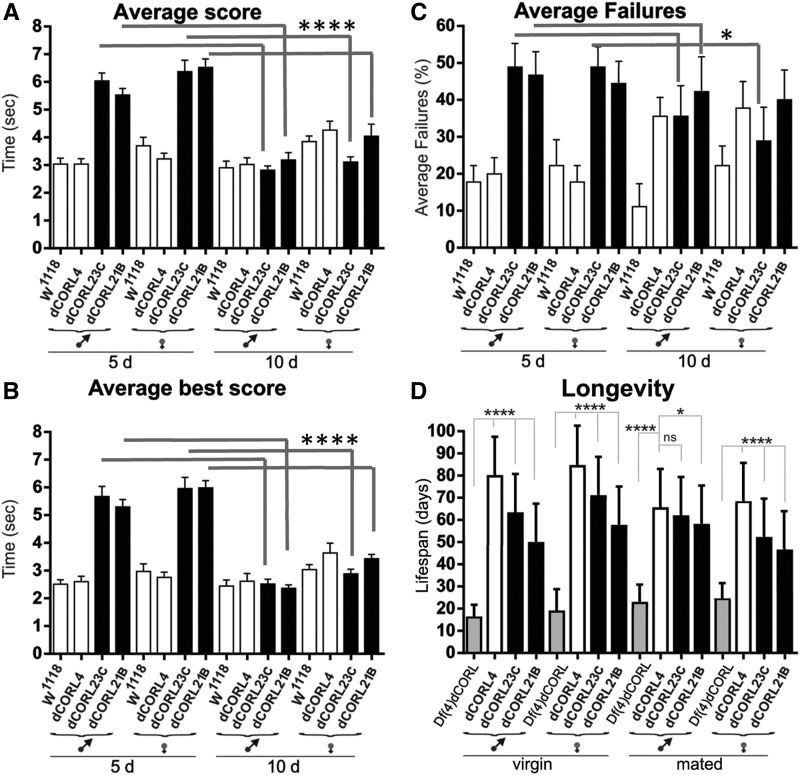
Quantification of age-dependent plasticity for climbing and longevity defects in *dCORL^23C^* and *dCORL^21B^*. A-C) Graphs from Fig. 6 reporting the analysis of age effects within each genotype, rather than comparisons between genotypes at the same age as before. Numerical data in Table S12. A) Average score shows that *dCORL^23C^* and *dCORL^21B^*males and females are significantly faster at 10 days than 5 days. *w^1118^* and *dCORL^4^* are unaffected by age or show declines in performance. B) Average best score shows the same results. *dCORL^23C^* and *dCORL^21B^* males and females are significantly faster at 10 days than 5 days while *w^1118^* and *dCORL^4^* are unaffected by age or show declines in performance. C) Average failure rate shows the same pattern with two differences. The significance of the improvement is not as robust as for climbing speed and *dCORL^21B^* females improve but not significantly. D) Longevity assays of virgin and mated males and females from *Df(4)dCORL*, *dCORL^4^*, *dCORL^23C^* and *dCORL^21B^*. Numerical data in Table S13. Lifespans of virgin and mated males and females from the three *dCORL* mutant strains are significantly shorter than *dCORL^4^* except *dCORL^21C^* mated males. *dCORL^23C^* and *dCORL^21B^* shortened lifespans do not show reductions of the magnitude of *Df(4)dCORL.*

We examined the effect of age within each genotype for these four strains, rather than between genotypes at the same age, with a goal of quantifying the age-dependent plasticity of each behavior for comparison to *Df(4)dCORL*. The analysis of average score and average best score showed that *dCORL^23C^* and *dCORL^21B^* males and females are significantly faster at 10 than 5 days, just like *Df(4)dCORL* (Figure 7A,B, numerical data in Table S12). The average failure rate also shows the same pattern with one difference, *dCORL^21B^* females improve but not significantly (Figure 7C). Alternatively, *Df(4)dCORL* females and males did not improve significantly, leading only to a match with *dCORL^21B^* females but not *dCORL^23C^* females or either set of males. Quantification shows that the distinction in the failure assay between *Df(4)dCORL* and the CRISPR mutants is one of degree, as all genotypes improved with age just not to exactly the same extent. The discrepancy does not negate the fact that the overall finding is similarity between *dCORL^23C^* and *dCORL^21B^* and *Df(4)dCORL*.

Lastly, we checked the longevity of virgin and mated males and females from *dCORL^23C^* and *dCORL^21B^* and *Df(4)dCORL* against the CRISPR control (Figure 7D). While the lifespans of all tested strains are significantly shorter than *dCORL^4^*, except *dCORL^21C^* mated males, neither of the *dCORL* CRISPR mutations showed reductions of the magnitude of *Df(4)dCORL*. Numerical data in Table S13. Quantification shows that the distinction in lifespan between *Df(4)dCORL* and the CRISPR mutants is one of degree. Thus, in virtually all assays the *dCORL^23C^* and *dCORL^21^*^B^ phenocopied *Df(4)dCORL* adding confidence to the association of *dCORL* loss with movement disorders displaying age-dependent plasticity.

## Discussion

How do the data impact our hypothesis that the association of *Fussel15* and *Fussel18* with ataxia will be conserved and visible in *dCORL* adult mutants as defects in movement-based behaviors? First, we are encouraged by the absence of a larval crawling phenotype for *Df(4)dCORL*. Most ataxias are not pediatric (Orr 2010) and thus the absence of a developmental component to the *Df(4)dCORL* adult climbing defect is consistent with the hypothesis. Second, the absence of any courtship defect attributable to *dCORL* in *Df(4)dCORL* adult males is not inconsistent with the hypothesis. The complex nature of courtship that includes modalities that are not movement-based such as olfaction and singing provide other potetial causes for this defect. In addition, *sphinx* and *Glu-RA* that lie within *Df(4)dCORL* have already been implicated in courtship (Dai *et al.* 2008, Chen *et al.* 2011; Schoenfeld *et al.* 2013). Third, the phototaxis defect attributable to *dCORL* is not caused by a photoreceptor defect and phototaxis is regulated by the same neurotransmitters that regulate movement - dopamine and octopamine (Gorostiza *et al.* 2016). This result is also consistent with our hypothesis.

Which brings us to the direct evidence in support of the hypothesis, our identification of adult climbing defects in two independenly generated *dCORL* mutants and their age-dependent plasticity. The defects in climbing are attributable to loss of *dCORL* due to assays with *dCORL^23C^* and *dCORL^21B^* and supported by the absence of climbing defects in six control strains with mutations in the region. We conclude that the hypothesis is true, *dCORL* mutations cause deficits in adult movement-based behaviors.

Given this, are there implications for *Fussel15* and *Fussel18* in our data? First to clarify, data from transgene assays in flies showed that mCORL2/SKOR2 and dCORL are both capable of fully rescuing an endogenous function in *Df(4)dCORL* larvae while mCORL1/SKOR1 cannot (Stinchfield *et al.* 2019). This fly transgene data indicates that movement defects in *dCORL* mutants are most relevant to *Fussel18/SKOR2* associated ataxias. Second, it is important to consider our observations of “attention-deficit” as the cause of the climbing and phototaxis defects. We noted that initiation of both behaviors appeared normal but then *Df(4)dCORL* flies would wander randomly. Third, the age-dependent increases in climbing speed that erase the initial deficits in average score and average best score in males and females of all *dCORL* mutant genotypes could reflect “hyperactivation” of the locomotor circuitry underlying walking. The last piece to consider is that the inbred spontaneously hypertensive rat is the most widely studied rodent model of Attention Deficit Hyperactivity Disorder (ADHD) and that these rats display both motor impairments and loss of Purkinje cells (Bruchhage *et al.* 2018). In the absence of a genetic model for ADHD, we propose that the *mCORL2/SKOR2* knockout mouse is a candidate. Further, the data suggest that polymorphisms in *Fussel18/SKOR2* be examined for association with ADHD in humans.

In an initial attempt to identify the mechanism behind the movement defects in *dCORL* mutants we pre-fed flies with yohimbine, an antagonist of tyramine receptors. In previous reports, yohimbine feeding rescued adult climbing and flight maintenance defects in a mutant characterized by low octopamine and high tyramine levels (Saraswati *et al.* 2004; Schützler *et al.* 2019). Tyramine is a metabolic precursor and functional antagonist of octopamine, the fly counterpart to norepinephrine in mammals. In each of the three climbing assays, the combination of age and yohimbine improved the scores of *Df(4)dCORL* flies over age alone. How *dCORL* might interact with a tyramine/octopamine locomotion circuit awaits further experimentation

Thinking broadly, a new hypothesis derived from the preliminary yohimbine data are that treatment of *Fussel18/SKOR2* associated ataxia with a norepinephrine antagonist would be therapeutic. A first step would be to test this hypothesis in *mCORL2/SKOR2* knockout mice with an ungainly gait from birth to adulthood (Wang *et al.* 2011). Rescue of the gait defect, analogous to the rescue of the climbing defects in *dCORL* mutants by age and yohimbine, is predicted if the hypothesis is true.

Overall, our data shows that *Df(4)dCORL* mutants exhibit three types of behavioral plasticity. First, significant climbing defects attributable to loss of *dCORL* are eliminated by age. Second, significant phototaxis defects due to loss of *dCORL* are partially ameliorated by age and are not due to faulty photoreceptors. Third, *Df(4)dCORL* males raised in groups have a lower courtship index than males raised as singles though this defect is not due to loss of *dCORL*. Subsequent tests showed that the climbing and phototaxis defects were phenocopied by *dCORL^21B^* and *dCORL^23C^* two CRISPR generated mutations. Overall, the finding that adult movement defects due to loss of *dCORL* are subject to age-dependent plasticity suggests new hypotheses for CORL functions in flies and mammals.
